# The Immunological Basis of Dry Eye Disease and Current Topical Treatment Options

**DOI:** 10.1089/jop.2019.0060

**Published:** 2020-04-07

**Authors:** Laura M. Periman, Victor L. Perez, Daniel R. Saban, Meng C. Lin, Piergiorgio Neri

**Affiliations:** ^1^ Periman Eye Center, Seattle, Washington.; ^2^ Duke Eye Center, Duke University School of Medicine, Durham, North Carolina.; ^3^ School of Optometry, Clinical Research Center, University of California, Berkeley, California.; ^4^ The Eye Institute, Cleveland Clinic Abu Dhabi, Abu Dhabi, United Arab Emirates.

**Keywords:** dry eye disease, ocular surface, inflammation, immunology, immune dysregulation, T cells, goblet cells, integrin protein, lymphocyte function-associated antigen 1 (LFA-1), intercellular adhesion molecule 1 (ICAM-1)

## Abstract

Homeostasis of the lacrimal functional unit is needed to ensure a well-regulated ocular immune response comprising innate and adaptive phases. When the ocular immune system is excessively stimulated and/or immunoregulatory mechanisms are disrupted, the balance between innate and adaptive phases is dysregulated and chronic ocular surface inflammation can result, leading to chronic dry eye disease (DED). According to the Tear Film and Ocular Surface Society Dry Eye Workshop II definition, DED is a multifactorial disorder of the ocular surface characterized by impairment and loss of tear homeostasis (hyperosmolarity), ocular discomfort or pain, and neurosensory abnormalities. Dysregulated ocular immune responses result in ocular surface damage, which is a further contributing factor to DED pathology. Several therapeutics are available to break the vicious circle of DED and prevent chronic disease and progression, including immunosuppressive agents (steroids) and immunomodulators (cyclosporine and lifitegrast). Given the chronic inflammatory nature of DED, each of these agents is commonly used in clinical practice. In this study, we review the immunopathology of DED and the molecular and cellular actions of current topical DED therapeutics to inform clinical decision making.

## Introduction

The Tear Film and Ocular Surface Society International Dry Eye Workshop II report defines dry eye disease (DED) as “a multifactorial disease of the ocular surface characterized by a loss of homeostasis of the tear film and accompanied by ocular symptoms, in which tear film instability and hyperosmolarity, ocular surface inflammation and damage, and neurosensory abnormalities play etiological roles.”^1^ DED is believed to be progressive in some patients.^2,3^ It can have significant impact on visual function, daily activities, social and physical functions, workplace productivity, and general quality of life.^4^ Global prevalence estimates range from 5% to 50%,^5^ and several reports find higher prevalence in women than in men.^6–8^ Additional risk factors include increasing age,^6–8^ systemic comorbidities such as diabetes and autoimmune disease,^9,10^ and therapeutic treatments for anxiety, depression, and sleep disorders.^11–15^ The pathologic processes of chronic inflammation and related biomarkers have been the focus of recent immunologic research to identify potential therapeutic targets.^16^ The “vicious cycle of inflammation” has been proposed as a core driver in DED with the bidirectional interaction between the ocular sensory neurons and local immune system disrupting ocular homeostasis.^16,17^ Ocular surface sensory neurons in response to inflammation can provoke nerve impulse activity resulting in differences in sensations, tear flow, and blinking.^17^ In addition, peripheral sensory neurons can illicit an immune response by releasing neuropeptides and immunomodulatory factors contributing to neurogenic inflammation.^17^ While there are many factors involved in DED such as/including hyperosmolarity, tear film instability, and meibomian gland dysfunction (MGD),^18,19^ the purpose of this article is to explore the specific immunopathogenic processes and the impact of current pharmacological therapies.

Over the past 15 years, a growing body of research on the role of inflammation in the pathogenesis of DED has led to the recognition of dysregulation of immune responses on the ocular surface.^20^ An appreciation of the basic immunological factors associated with DED is essential for appropriate management of patients with the disease.^21^ At the most basic level, there are 2 phases of immune responses coordinated to provide protection: innate and adaptive. Innate immune responses, which occur at the ocular surface, provide a first-line generalized defense.^22^ Irritation, such as environmental or endogenous factors, or microbial stress on the ocular surface, activates signaling pathways that initiate an acute inflammatory response, including stimulation of the production of pro-inflammatory cytokines, matrix metalloproteinases (MMPs), and chemokines. The more specific adaptive immune response is then signaled to produce antigen-specific T cells in regional lymph nodes that migrate to the ocular surface to respond to the ocular stressors. DED in the chronic adaptive immunity phase is, in effect, a locoregional disease. The proliferation of T cells in the adaptive immunity phase and their amplified activation at the ocular surface cause damage that reinitiates the acute pro-inflammatory innate response, which, when accompanied by loss of immunoregulation, triggers a vicious circle^23^ of pathological immune response.

Although the innate and adaptive immune responses differ and are triggered in distinct regions, key molecular interactions facilitating cellular migration are common to both. The interactions between 2 cell-surface factors—lymphocyte function-associated antigen 1 (LFA-1, an integrin protein) and its associated ligand, intercellular adhesion molecule 1 (ICAM-1)—are key to the proliferation and infiltration of immune cells. Basic knowledge of their roles in the dysregulation of ocular immunity is key to understanding current patient treatment strategies for DED.

## The Ocular Surface Immune Response and DED

The ocular surface immune response involves both innate and adaptive mechanisms.^24^ It occurs at the corneal surface, in ocular tissues and regional lymph nodes, and involves T helper (T_H_) cells, memory T cells, and regulatory T cells (Tregs).^25^ It is a complex and tightly regulated process that is designed to protect and defend the ocular surface but, when dysregulated, can lead to DED.^19,26–29^

Specific insults or stress to the corneal surface triggers an innate immune response on the ocular surface that is maintained and regulated by the corneal epithelium. Mitogen-activated protein kinases (MAP-Ks)—specifically c-Jun N-terminal kinase (JNK), extracellular signal–related kinase (ERK), and p38—are activated and stimulate transcription nuclear factor kappa B (NF-κB), chemokines, and MMPs.^30,31^ A variety of immune cells reside at the ocular surface: natural killer immune cells, dendritic cells (which are the primary antigen-presenting cells [APCs]), macrophages, gamma delta (γδ) cells, and, to a limited extent, alpha beta (αβ) T cells (CD4^+^ and CD8^+^).^26,32,33^ During the innate response, specific immune cells at the ocular surface are activated to respond to the insult.^4^ Cytokines and chemokines, specifically tumor necrosis factor alpha (TNF-α) and interleukin 1 and 6 (IL-1, IL-6), stimulate the maturation of APCs, while chemokine receptor 7 (CCR7) facilitates migration of mature APCs (mAPCs) in the afferent lymphatic vessels.^33,34^ These mAPCs are the primary immune cells that bridge the innate and adaptive immune responses.^33^

In the adaptive immune response, mAPCs that have migrated to regional lymph nodes through the afferent arm facilitate the differentiation of naive T (T_H_0) cells into several types of mature T cells, such as (1) memory T cells, unique and specific to the antigen that caused the insult; (2) T_H_ cells, which become the circulating effector T cells^35^; and (3) Tregs, which modulate the immune response.^33^ All of these T cells are generated in the lymph nodes and subsequently migrate to the site of inflammation, the conjunctiva, and the ocular surface.^35–37^ Although these have seemingly contradictory functions, they all play a role in a normal response, highlighting the multiplicities and complexities of the immune system.

Memory T cells may survive in an inactive state for long periods and proliferate upon restimulation (possibly at the ocular surface).^35,38^ T_H_ cells can be autoreactive—that is, they can react to self-antigens.^35^ Once activated, they secrete pro-inflammatory factors that sustain the immune response.^27,39^ Loss of homeostatic mechanisms of the lacrimal functional unit can lead to dysregulation of the natural immune responses.^40^ Myriad systemic conditions are associated with loss of homeostatic mechanisms, including hormonal abnormalities, systemic and topical medications, preexisting ocular stress, lifestyle habits, and environmental conditions.^5,18,41,42^

**FIG. 1.**
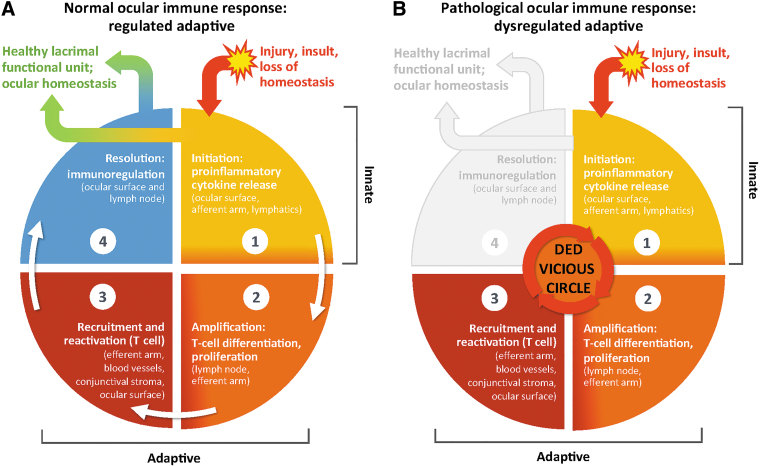
Ocular surface immune response. **(A)** A healthy system can respond to injury and invaders by mounting a response, eliminating the pathogens while limiting tissue damage, then returning to homeostasis. **(B)** DED is a chronic worsening spiral of immune response with loss of return to homeostasis (a vicious *circle*^23^ leading to damage and self-perpetuation). DED, dry eye disease.

Inflammation at the ocular surface can be both a cause and a consequence of DED.^23^ The resolution of inflammation at the ocular surface is usually controlled by immunoregulatory processes, such as goblet cell secretion of the immunoregulatory cytokine transforming growth factor beta (TGF-β) and programmed death-ligand 1 regulation of activated effector T cells.^35,40^ When homeostatic control mechanisms fail and immunoregulatory mechanisms such as these are suppressed or overwhelmed, the immune response becomes amplified, particularly the adaptive response. This results in increased mAPC activity and increased production and recruitment of CD4^+^ T_H_ cells to the ocular surface. At the ocular surface, the dysregulated activity of effector T cells includes increased release of pro-inflammatory cytokines, causing further inflammation and damage.^39,43^ This, in turn, reinitiates the innate immune response, thus creating a vicious circle.^23^ The resolution of inflammation by immunoregulatory processes is bypassed and/or inadequate to address the sustained inflammation (Fig. 1).^35,40^

## Immunopathogenesis of DED

**FIG. 2.**
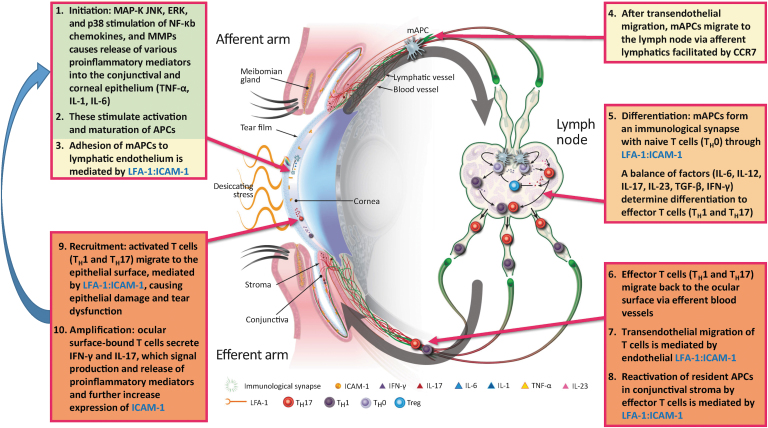
The immunoinflammatory pathway of dry eye disease. APC, antigen-presenting cell; CCR, chemokine receptor; ERK, extracellular signal–related kinase; ICAM-1, intercellular adhesion molecule 1; IFN-γ, interferon gamma; IL-, interleukin; JNK, c-Jun N-terminal kinase; LFA-1, lymphocyte function-associated antigen 1; mAPC, mature antigen-presenting cell; MAP-K, mitogen-activated protein kinase; MMP, matrix metalloproteinase; NF-κB, nuclear factor kappa B; TGF-β, transforming growth factor beta; TNF-α, tumor necrosis factor alpha; Treg, regulatory T cell. Adapted from Pflugfelder et al.^52^

**FIG. 3.**
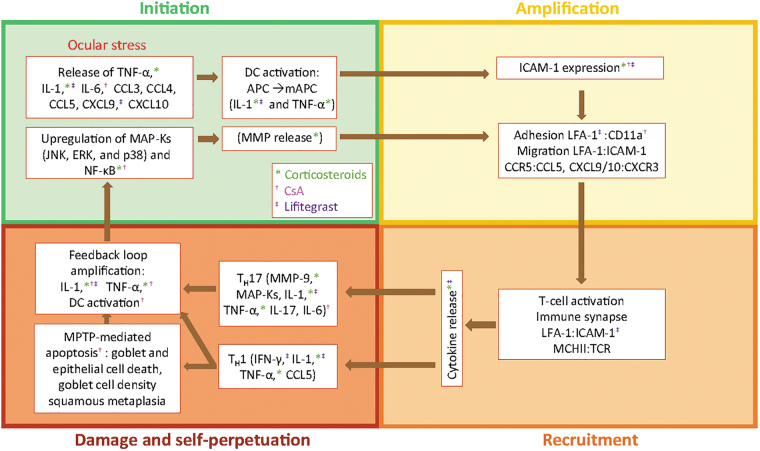
The phases of DED pathology and its processes, effectors, and actual treatments. Color-coded symbols denote specific DED treatment (corticosteroids, CsA, lifitegrast) modulation of DED effectors. APC, antigen-presenting cell; CCL, CXCL, chemokines; CCR, CXCR, chemokine receptors; CD11a, cluster of designation molecule 11a; CsA, cyclosporin A; DC, dendritic cell; DED, dry eye disease; ERK, extracellular signal–related kinase; ICAM-1, intercellular adhesion molecule 1; IFN-γ, interferon gamma; IL, interleukin; JNK, c-Jun N-terminal kinase; LFA-1, lymphocyte function-associated antigen 1; MAP-K, mitogen-activated protein kinase; mAPC, mature antigen-presenting cell; MHCII, major histocompatibility complex II; MMP, matrix metalloproteinase; MPTP, 1-methyl-4-phenyl-1,2,3,6-tetrahydropyridine; NF-κB, nuclear factor kappa B; TCR, T cell receptor; TNF-α, tumor necrosis factor alpha.

Broken down stepwise, the immunopathogenesis of DED is a 4-part process of initiation, amplification, recruitment, and damage/self-perpetuation (Figs. 2 and 3). The initiation phase of the innate immune response at the ocular surface induces localized acute inflammation.^27^ Specifically, initiation involves upregulation of MAP-K JNK, ERK, and p38 stimulation of NF-κB. Various pro-inflammatory mediators, including cytokines (TNF-α, IL-1, IL-6), various inflammatory chemokines (CCL3, CCL4), and T cell–attracting chemokines (CCL5, CXCL9, CXCL10), are released into the corneal and conjunctival epithelium (Fig. 2: step 1).^30,31^ The release of IL-1 and TNF-α induces the activation and maturation of APCs, which are predominantly antigen-presenting dendritic cells (Fig. 2: step 2).^34^ Upregulated ICAM-1 on lymphatic endothelial cells facilitates mAPC adhesion (Fig. 2: step 3).^44,45^ The mAPCs then migrate, in a CCR7-mediated manner, through the efferent arm of the ocular anatomy to regional lymph nodes (Fig. 2: step 4).^46–48^ The adaptive immune response involves the creation and recruitment of effector T cells.^49,50^ This occurs in the following manner: in the lymph node, mAPCs engage with T_H_0 cells through an immune synapse (partly mediated by LFA-1:ICAM-1 interaction and also T cell receptor and major histocompatibility complex, TCR:MHC) to cause differentiation of T_H_0 cells into several subsets of T_H_ cells: T_H_1, T_H_2,T_H_17, and Tregs. T cell differentiation is determined by mAPC expression of a balance of signaling factors, including IL-6, IL-12, IL-17, IL-23, TGF-β, and interferon gamma (IFN-γ) (Fig. 2: step 5).^51^ The proportions and balance of the various cytokines help to drive differentiation of the effector T cells into specific subtypes.

Effector T cells then migrate through the efferent arm blood vessels to the conjunctival stroma (Fig. 2: steps 6 and 7).^44,45,52–54^ There, they reactivate resident mAPCs and are recruited to the ocular surface (Fig. 2: steps 8 and 9).^40^ Specifically, T_H_1 and T_H_17 are the primary lymphocyte cells involved in the ocular surface damage and inflammation that is related to DED.^35^ They release cytokines that alter the normal balance and cause epithelial damage and tear dysfunction.^55,56^ This, in turn, elicits an immune response and fuels the self-perpetuation cycle of DED pathogenesis (Fig. 2: step 10).^18^ In nonpathogenic responses, the Tregs are responsible for dampening the effector response and regulating immunity.^55^

T cell infiltration of the lacrimal gland and conjunctiva and amplification of the release of inflammatory cytokines are features of chronic inflammation in DED.^23,27,40,57^ Pro-inflammatory T_H_1 cells secrete the hallmark cytokine IFN-γ, which has been shown to promote conjunctival goblet cell loss and apoptosis of the ocular surface epithelium.^37,58^ In addition, IFN-γ is associated with conjunctival squamous epithelial hyperplasia.^37^ T_H_17 cells secrete the hallmark cytokine IL-17 and promote production of the MMPs from corneal epithelial cells and fibroblasts.^31,59,60^ MMPs contribute to disruption of corneal epithelial barrier function, which contributes to further ocular insults.^26^ IFN-γ and IL-17 also exert pathogenic effects by promoting production of pro-inflammatory cytokines, chemokines, MMPs, cell adhesion molecules, and prolymphangiogenic molecules.^40^ These modulators lead to further damage of the ocular surface and thus amplify the cycle of inflammation.

## Molecular Interactions Key to DED Pathogenesis

Integrins are cell-surface proteins expressed on a variety of cells. They are upregulated in response to insults and also play key roles in integrating signals among various cells.^28,54,61^ The integrin LFA-1 is a leukocyte cell-surface glycoprotein and a modulator of T cell activation and proliferation.^62^ LFA-1 on T cells exists in an inactive or low-affinity binding state (bent conformation), but undergoes a conformational change to the high-affinity form of LFA-1 in response to inflammation. Specifically, this conformational change is due to TCR engagement in T cells and CXCR2 in neutrophils.^63^ ICAM-1 is the natural ligand of LFA-1. During inflammation, acute-phase cytokines IL-1 and TNF-α induce upregulation of ICAM-1 expression on a variety of cells, including the vascular endothelium of patients with DED.^36,44,64–66^ Upregulation of ICAM-1 has several important functions, described further below (Fig. 2).^67^

The LFA-1:ICAM-1 interaction has several important roles at key points in the immunoinflammatory pathway of DED (Fig. 2).^52^ In general, binding of dendritic cells to the vascular endothelium through LFA-1:ICAM-1 interactions facilitates their migration to regional lymph nodes.^25^ LFA-1:ICAM-1 interactions help to form the immune synapse between mAPCs and T_H_0 cells, leading to T cell differentiation.^61^ The LFA-1:ICAM-1 interaction also aids the migration of activated T cells from blood vessels to the site of inflammation.^45^ LFA-1:ICAM-1 interactions are thought to be necessary for the activation of effector T cells at the ocular surface through the immune synapse.^24^ LFA-1:ICAM-1 interactions also play a role in the recruitment of T cells at the conjunctival epithelium and ocular surface.^4^ Finally, the release of cytokines and chemokines is signaled through immune synapse/dual interactions between T cells and mAPCs using LFA-1:ICAM-1 and TCR:MHC, respectively.^24,52–54^ As the LFA-1:ICAM-1 interaction has a major role in ocular surface inflammation and the ocular immune response, treatment strategies focused on their association have emerged as therapeutic targets.^52^ Whatever the precipitating cause of a patient's DED, treatment options are required that disrupt the chronic inflammatory process.^20,21^

## Topical Ophthalmic Treatments for DED

Topical treatments for DED that are currently available in the United States and Canada include immunosuppressive agents (corticosteroids), immunomodulatory agents (cyclosporine ophthalmic emulsion 0.05% and cyclosporine ophthalmic solution 0.09%), and the recently available LFA-1 antagonist lifitegrast ophthalmic solution 5%.^68^ The factors and processes involved in DED pathology, and the DED treatments that modulate these factors and processes, are summarized in Figure 3.

As potent inhibitors of multiple inflammatory mediators, topical corticosteroids are effective in interrupting the cycle of inflammation.^37^ Suppression of NF-κB leads to suppression of acute-phase cytokines IL-1 and TNF-α, ICAM-1, MMPs, cytokines, chemokines, prostaglandins, and phospholipase A.^65,69,70^ Topical corticosteroids also reduce leukocyte infiltration of inflamed ocular tissues.^71,72^ However, known side effects (intraocular hypertension, cataracts, decreased wound healing, and predisposition to infection) limit their long-term use.^72^ KPI-121, an investigational nanotechnology-based formulation of the corticosteroid loteprednol, has been evaluated in recently completed phase 3 clinical trials for patients with DED (NCT02793817 and NCT02813265); imminent publication of the results is expected.^73^

Topical cyclosporin A (CsA) is indicated to increase tear production in patients with keratoconjunctivitis sicca.^74^ CsA works by inhibiting the calcineurin–phosphatase pathway by intracellular complex formation with cyclophilin.^75^ The clinical mechanism of action of CsA has not been fully elucidated,^74^ but it includes increased natural tear production and increased goblet cell density.^38,76^ The scientific literature reports numerous molecular effects of CsA on DED immunopathophysiology, including inhibited T cell activation,^74^ decreased cyclophilin-mediated gene transcription of IL-2 and IL-6,^39^ and decreased epithelial and goblet cell apoptosis.^77^

CsA inhibits the activation of T cells as measured by immunoactivation markers of human leukocyte antigen-D related and cluster of designation molecule 11a (CD11a) cell counts.^76^ CD11a is a subunit of LFA-1.^75,78^ However, T cells that have already been activated can live for up to 164 days.^79^

The original phase 3 clinical trial of CsA in patients with moderate-to-severe DED symptoms reported statistically significant improvements in subjective symptoms (*P* < 0.05), such as blurred vision, at 4 weeks. This was consistent with the improvements seen in objective signs: in corneal staining at 4 months and categorized Schirmer values (improved tear production) at 6 months.^80^ The most common side effect of CsA treatment was ocular burning.^81^ Other side effects included blurred vision, ocular itching, conjunctival hyperemia, discharge, foreign body sensation, and stinging.^74,81^ CsA ophthalmic solution 0.09%, recently approved in the United States, also showed increased tear production of ≥10 mm by Schirmer's test after 12 weeks of treatment compared with vehicle (16.8% vs. 8.6%, respectively).^82^ The most common adverse event was mild, moderate, or severe instillation-site pain (the majority were mild).^82^ Additional side effects included conjunctival hyperemia, blepharitis, eye irritation, headache, and urinary tract infection.^82^ The exact molecular and cellular mechanism of action of this new CsA concentration and formulation remains to be elucidated.

**FIG. 4.**
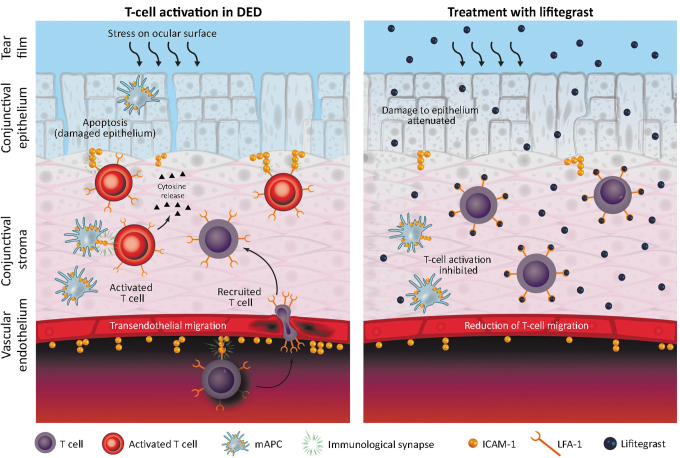
Effect of allosteric binding of lifitegrast to LFA-1 on LFA-1:ICAM-1 interactions. DED, dry eye disease; ICAM-1, intercellular adhesion molecule 1; LFA-1, lymphocyte function associated antigen 1; mAPC, mature antigen-presenting cell. Adapted from Perez et al.^25^

Lifitegrast is approved in North America for the treatment of the signs and symptoms of DED in adult patients.^83^ It is an LFA-1 antagonist with a proposed mechanism of action of specifically blocking the binding of ICAM-1 to LFA-1 with high affinity (Fig. 4).^25^ Lifitegrast has the potential to act on both afferent and efferent arms of the immunomodulatory pathway in DED.^84^ In the afferent arm (to the lymph node), it may block LFA-1:ICAM-1 interaction between dendritic cells on the ocular surface and endothelial cells of lymphatic tissues, thereby inhibiting migration and homing of naive dendritic cells to draining lymph nodes or activation of resting T cells at the ocular surface.^25^ In the efferent arm (from the lymph node), lifitegrast may inhibit migration of activated T cells into the conjunctiva, recruitment in conjunctival epithelium, and secondary activation in ocular tissues.^52^ In experimental models, lifitegrast inhibited IFN-γ, IL-1β, IL-10, and macrophage inflammatory protein 1 alpha levels.^85^

A recent lifitegrast study in a murine model of desiccating stress provided evidence of lower expression of IFN-γ, CXCL9, and T_H_1-related genes compared with controls.^86^ Mice treated with lifitegrast also had less corneal barrier disruption and greater conjunctival goblet cell density compared with controls.^86^ Results of *in vivo* studies of lifitegrast in mice and dogs have demonstrated a potent dose-dependent inhibition of T cell activation, T cell recruitment, and release of cytokines that has been shown to correlate with clinical severity of DED.^85,87–89^ Likewise, in the chronic allergic eye disease murine model, an immune-mediated form of MGD is induced through a surprising T_H_17 and polymorphonuclear neutrophil response and has clinical parallels in humans.^90^ Subsequent studies with lifitegrast treatment in this murine allergic eye disease model resulted in reduced severity of MGD and were associated with reduced Th17 cells and polymorphonuclear neutrophils in the conjunctiva (Saban DR. 2019; article in preparation). Clinical studies with lifitegrast have demonstrated improvements in signs and symptoms of DED.^91–94^ Combined results from phase 3 efficacy and safety studies (OPUS-1,^92^ OPUS-2,^91^ and OPUS-3^93^) and a 1-year safety study (SONATA^94^) demonstrated that lifitegrast alleviated symptoms of DED, with rapid onset of effect and improved signs of DED. The most common treatment-emergent adverse event was altered taste sensation (dysgeusia).^95^

Additional anti-inflammatory therapies for the treatment of DED are under investigation. RGN-259 ophthalmic solution is a synthetic copy of the naturally occurring 43 amino acid protein thymosin β4 (Tβ4), which is the major constituent protein of trauma response and wound repair cells such as platelets, macrophages, and polymorphonuclear cells.^96^ Tβ4 was found to promote corneal epithelial cell migration, decrease inflammation, and accelerate epithelialization in a murine-controlled adverse environment model of DED.^97^ In a phase 2 trial with RGN-259, the combined primary end point of signs and symptoms of DED was not met.^97^ However, multiple secondary end points showed improvements with RGN-259 in the phase 2 trial and a recently completed phase 3 trial (NCT02974907).^97–100^

## Conclusion

DED is a multifactorial disease that is characterized by a sustained inflammatory response on the ocular surface that, if left untreated, can lead to chronic disease. Whether DED can be considered “progressive”—that is, whether any clinical characteristics of DED will progress without treatment—remains to be determined. Progress in discerning the underlying immunopathology of DED has led to advances in treatment that target specific inflammatory effectors/pathways. Such treatments are needed to break the cycle of DED and prevent chronic disease and progression.

### Method of Literature Search

PubMed searches were performed and included articles from 1987 through 2018. Terms related to dry eye (“dry eye,” “dry eye disease,” “keratoconjunctivitis sicca,” “DED”) and the ocular immune response (“immune response,” “immunopathology,” “pathophysiology,” “inflammation,” “ocular surface immune response”) were included. Related searches on specific ocular immune effectors, including “T cell,” “dendritic cell,” “goblet cell,” “integrin protein,” “adhesion molecule,” “lymphocyte function-associated antigen 1,” “LFA-1,” “intercellular adhesion molecule 1,” “ICAM-1,” and specific topics regarding DED prevalence and DED-associated comorbidities were also conducted in PubMed and Google. English translations of abstracts were reviewed from all languages. Articles were read from English sources. From the abstracts, articles were reviewed that addressed areas of ocular surface pathology, immunology, inflammation, and associated treatments of DED.

## References

[1] Craig J.P., Nichols K.K., Akpek E.K., Caffery B., Dua H.S., Joo C.-K., Liu Z., Nelson J.D., Nichols J.J., Tsubota K., and Stapleton F. TFOS DEWS II definition and classification report. Ocul. Surf. 15:276–283, 2017 28736335 10.1016/j.jtos.2017.05.008

[2] Lemp M.A., Crews L.A., Bron A.J., Foulks G.N., and Sullivan B.D. Distribution of aqueous-deficient and evaporative dry eye in a clinic-based patient cohort: a retrospective study. Cornea. 31:472–478, 2012 22378109 10.1097/ICO.0b013e318225415a

[3] Stern M.E., Gao J., Siemasko K.F., Beuerman R.W., and Pflugfelder S.C. The role of the lacrimal functional unit in the pathophysiology of dry eye. Exp. Eye Res. 78:409–416, 2004 15106920 10.1016/j.exer.2003.09.003

[4] Pflugfelder S.C. Prevalence, burden, and pharmacoeconomics of dry eye disease. Am. J. Manag. Care. 14 Suppl 3:S102–S106, 2008 18452369

[5] Stapleton F., Alves M., Bunya V.Y., Jalbert I., Lekhanont K., Malet F., Na K.-S., Schaumberg D., Uchino M., Vehof J., Viso E., Vitale S., and Jones L. TFOS DEWS II Epidemiology Report. Ocul. Surf. 15:334–365, 2017 28736337 10.1016/j.jtos.2017.05.003

[6] Paulsen A.J., Cruickshanks K.J., Fischer M.E., Huang G.-H., Klein B.E.K., Klein R., and Dalton D.S. Dry eye in the Beaver Dam Offspring Study: prevalence, risk factors, and health-related quality of life. Am. J. Ophthalmol. 157:799–806, 2014 24388838 10.1016/j.ajo.2013.12.023 PMC3995164

[7] Moss S.E., Klein R., and Klein B.E.K. Prevalence of and risk factors for dry eye syndrome. Arch. Ophthalmol. 118:1264–1268, 2000 10980773 10.1001/archopht.118.9.1264

[8] Schaumberg D.A., Uchino M., Christen W.G., Semba R.D., Buring J.E., and Li J.Z. Patient reported differences in dry eye disease between men and women: impact, management, and patient satisfaction. PLoS One. 8:e76121, 2013 24098772 10.1371/journal.pone.0076121 PMC3786885

[9] Wang T.-J., Wang I.-J., Hu C.-C., and Lin H.-C. Comorbidities of dry eye disease: a nationwide population-based study. Acta Ophthalmol. 90:663–668, 2012 20809911 10.1111/j.1755-3768.2010.01993.x

[10] Roh H.C., Lee J.K., Kim M., Oh J.-H., Chang M.-W., Chuck R.S., and Park C.Y. Systemic comorbidities of dry eye syndrome: the Korean National Health and Nutrition Examination Survey V, 2010 to 2012. Cornea. 35:187–192, 2016 26488632 10.1097/ICO.0000000000000657

[11] Wong J., Lan W., Ong L.M., and Tong L. Non-hormonal systemic medications and dry eye. Ocul. Surf. 9:212–226, 2011 22023816 10.1016/s1542-0124(11)70034-9

[12] van der Vaart R., Weaver M.A., Lefebvre C., and Davis R.M. The association between dry eye disease and depression and anxiety in a large population-based study. Am. J. Ophthalmol. 159:470–474, 2015 25461298 10.1016/j.ajo.2014.11.028 PMC4329250

[13] Szakáts I., Sebestyén M., Németh J., Birkás E., and Purebl G. The role of health anxiety and depressive symptoms in dry eye disease. Curr. Eye Res. 41:1044–1049, 2016 26642862 10.3109/02713683.2015.1088955

[14] Na K.-S., Han K., Park Y.-G., Na C., and Joo C.-K. Depression, stress, quality of life, and dry eye disease in Korean women: a population-based study. Cornea. 34:733–738, 2015 26002151 10.1097/ICO.0000000000000464

[15] Koçer E., Koçer A., Özsütçü M., Dursun A.E., and Kirpinar I. Dry eye related to commonly used new antidepressants. J. Clin. Psychopharmacol. 35:411–413, 2015 26075491 10.1097/JCP.0000000000000356

[16] Yamaguchi T. Inflammatory Response in Dry Eye. Invest. Ophthalmol. Vis. Sci. 59:Des192-des199, 2018 30481826 10.1167/iovs.17-23651

[17] Belmonte C., Nichols J.J., Cox S.M., Brock J.A., Begley C.G., Bereiter D.A., Dartt D.A., Galor A., Hamrah P., Ivanusic J.J., Jacobs D.S., McNamara N.A., Rosenblatt M.I., Stapleton F., and Wolffsohn J.S. TFOS DEWS II pain and sensation report. Ocul. Surf. 15:404–437, 2017 28736339 10.1016/j.jtos.2017.05.002 PMC5706540

[18] Bron A.J., de Paiva C.S., Chauhan S.K., Bonini S., Gabison E.E., Jain S., Knop E., Markoulli M., Ogawa Y., Perez V., Uchino Y., Yokoi N., Zoukhri D., and Sullivan D.A. TFOS DEWS II Pathophysiology Report. Ocul. Surf. 15:438–510, 2017 28736340 10.1016/j.jtos.2017.05.011

[19] Pflugfelder S.C., and de Paiva C.S. The pathophysiology of dry eye disease: what we know and future directions for research. Ophthalmology. 124:S4–S13, 2017 29055361 10.1016/j.ophtha.2017.07.010 PMC5657523

[20] Wei Y., and Asbell P.A. The core mechanism of dry eye disease is inflammation. Eye Contact Lens. 40:248–256, 2014 25390549 10.1097/ICL.0000000000000042 PMC4231828

[21] Yagci A., and Gurdal C. The role and treatment of inflammation in dry eye disease. Int. Ophthalmol. 34:1291–1301, 2014 25416345 10.1007/s10792-014-9969-x

[22] Bolaños-Jiménez R., Navas A., López-Lizárraga E.P., de Ribot F.M., Peña A., Graue-Hernández E.O., and Garfias Y. Ocular surface as barrier of innate immunity. Open Ophthalmol. J. 9:49–55, 2015 26161163 10.2174/1874364101509010049 PMC4484240

[23] Baudouin C. The pathology of dry eye. Surv. Ophthalmol. 45 Suppl 2:S211–S220, 2001 11587145 10.1016/s0039-6257(00)00200-9

[24] Schaumburg C.S., Siemasko K.F., De Paiva C.S., Wheeler L.A., Niederkorn J.Y., Pflugfelder S.C., and Stern M.E. Ocular surface APCs are necessary for autoreactive T cell-mediated experimental autoimmune lacrimal keratoconjunctivitis. J. Immunol. 187:3653–3662, 2011 21880984 10.4049/jimmunol.1101442

[25] Perez V.L., Pflugfelder S.C., Zhang S., Shojaei A., and Haque R. Lifitegrast, a novel integrin antagonist for treatment of dry eye disease. Ocul. Surf. 14:207–215, 2016 26807723 10.1016/j.jtos.2016.01.001

[26] Zhang X., Volpe E.A., Gandhi N.B., Schaumburg C.S., Siemasko K.F., Pangelinan S.B., Kelly S.D., Hayday A.C., Li D.-Q., Stern M.E., Niederkorn J.Y., Pflugfelder S.C., and De Paiva C.S. NK cells promote Th-17 mediated corneal barrier disruption in dry eye. PLoS One. 7:e36822, 2012 22590618 10.1371/journal.pone.0036822 PMC3348128

[27] Stern M.E., Schaumburg C.S., Siemasko K.F., Gao J., Wheeler L.A., Grupe D.A., De Paiva C.S., Calder V.L., Calonge M., Niederkorn J.Y., and Pflugfelder S.C. Autoantibodies contribute to the immunopathogenesis of experimental dry eye disease. Invest. Ophthalmol. Vis. Sci. 53:2062–2075, 2012 22395876 10.1167/iovs.11-9299 PMC6713471

[28] Bertoni A., Alabiso O., Galetto A.S., and Baldanzi G. Integrins in T cell physiology. Int. J. Mol. Sci. 19:485, 2018 10.3390/ijms19020485 PMC5855707 29415483

[29] Barbosa F.L., Xiao Y., Bian F., Coursey T.G., Ko B.Y., Clevers H., de Paiva C.S., and Pflugfelder S.C. Goblet cells contribute to ocular surface immune tolerance—implications for dry eye disease. Int. J. Mol. Sci. 18:978, 2017 10.3390/ijms18050978 PMC5454891 28475124

[30] Li D.-Q., Luo L., Chen Z., Kim H.S., Song X.J., and Pflugfelder S.C. JNK and ERK MAP kinases mediate induction of IL-1β, TNF-α and IL-8 following hyperosmolar stress in human limbal epithelial cells. Exp. Eye Res. 82:588–596, 2006 16202406 10.1016/j.exer.2005.08.019 PMC2198933

[31] Luo L., Li D.-Q., Doshi A., Farley W., Corrales R.M., and Pflugfelder S.C. Experimental dry eye stimulates production of inflammatory cytokines and MMP-9 and activates MAPK signaling pathways on the ocular surface. Invest. Ophthalmol. Vis. Sci. 45:4293–4301, 2004 15557435 10.1167/iovs.03-1145

[32] Strauss-Albee D.M., Horowitz A., Parham P., and Blish C.A. Coordinated regulation of NK receptor expression in the maturing human immune system. J. Immunol. 193:4871–4879, 2014 25288567 10.4049/jimmunol.1401821 PMC4225175

[33] Stevenson W., Chauhan S.K., and Dana R. Dry eye disease: an immune-mediated ocular surface disorder. Arch. Ophthalmol. 130:90–100, 2012 22232476 10.1001/archophthalmol.2011.364 PMC3677724

[34] Hamrah P., Liu Y., Zhang Q., and Dana M.R. Alterations in corneal stromal dendritic cell phenotype and distribution in inflammation. Arch. Ophthalmol. 121:1132–1140, 2003 12912691 10.1001/archopht.121.8.1132

[35] El Annan J., Chauhan S.K., Ecoiffier T., Zhang Q., Saban D.R., and Dana R. Characterization of effector T cells in dry eye disease. Invest. Ophthalmol. Vis. Sci. 50:3802–3807, 2009 19339740 10.1167/iovs.08-2417 PMC2921683

[36] Stern M.E., Gao J., Schwalb T.A., Ngo M., Tieu D.D., Chan C.C., Reis B.L., Whitcup S.M., Thompson D., and Smith J.A. Conjunctival T-cell subpopulations in Sjögren's and non-Sjögren's patients with dry eye. Invest. Ophthalmol. Vis. Sci. 43:2609–2614, 2002 12147592

[37] De Paiva C.S., Villarreal A.L., Corrales R.M., Rahman H.T., Chang V.Y., Farley W.J., Stern M.E., Niederkorn J.Y., Li D.-Q., and Pflugfelder S.C. Dry eye–induced conjunctival epithelial squamous metaplasia is modulated by interferon-γ. Invest. Ophthalmol. Vis. Sci. 48:2553–2560, 2007 17525184 10.1167/iovs.07-0069

[38] Kunert K.S., Tisdale A.S., and Gipson I.K. Goblet cell numbers and epithelial proliferation in the conjunctiva of patients with dry eye syndrome treated with cyclosporine. Arch. Ophthalmol. 120:330–337, 2002 11879137 10.1001/archopht.120.3.330

[39] Stern M.E., Schaumburg C.S., Dana R., Calonge M., Niederkorn J.Y., and Pflugfelder S.C. Autoimmunity at the ocular surface: pathogenesis and regulation. Mucosal Immunol. 3:425–442, 2010 20485329 10.1038/mi.2010.26 PMC3577924

[40] Barabino S., Chen Y., Chauhan S., and Dana R. Ocular surface immunity: homeostatic mechanisms and their disruption in dry eye disease. Prog. Retin. Eye Res. 31:271–285, 2012 22426080 10.1016/j.preteyeres.2012.02.003 PMC3334398

[41] Sullivan D.A., Rocha E.M., Aragona P., Clayton J.A., Ding J., Golebiowski B., Hampel U., McDermott A.M., Schaumberg D.A., Srinivasan S., Versura P., and Willcox M.D.P. TFOS DEWS II Sex, Gender, and Hormones Report. Ocul. Surf. 15:284–333, 2017 28736336 10.1016/j.jtos.2017.04.001

[42] Gomes J.A.P., Azar D.T., Baudouin C., Efron N., Hirayama M., Horwath-Winter J., Kim T., Mehta J.S., Messmer E.M., Pepose J.S., Sangwan V.S., Weiner A.L., Wilson S.E., and Wolffsohn J.S. TFOS DEWS II Iatrogenic Report. Ocul. Surf. 15:511–538, 2017 28736341 10.1016/j.jtos.2017.05.004

[43] Stern M.E., Schaumburg C.S., and Pflugfelder S.C. Dry eye as a mucosal autoimmune disease. Int. Rev. Immunol. 32:19–41, 2013 23360156 10.3109/08830185.2012.748052 PMC3587314

[44] Johnson L.A., Clasper S., Holt A.P., Lalor P.F., Baban D., and Jackson D.G. An inflammation-induced mechanism for leukocyte transmigration across lymphatic vessel endothelium. J. Exp. Med. 203:2763–2777, 2006 17116732 10.1084/jem.20051759 PMC2118156

[45] Muller W.A. Getting leukocytes to the site of inflammation. Vet. Pathol. 50:7–22, 2013 23345459 10.1177/0300985812469883 PMC3628536

[46] Schlereth S., Lee H.S., Khandelwal P., and Saban D.R. Blocking CCR7 at the ocular surface impairs the pathogenic contribution of dendritic cells in allergic conjunctivitis. Am. J. Pathol. 180:2351–2360, 2012 22507838 10.1016/j.ajpath.2012.02.015 PMC5691338

[47] Saban D.R. The chemokine receptor CCR7 expressed by dendritic cells: a key player in corneal and ocular surface inflammation. Ocul. Surf. 12:87–99, 2014 24725321 10.1016/j.jtos.2013.10.007 PMC3986807

[48] Kodati S., Chauhan S.K., Chen Y., Dohlman T.H., Karimian P., Saban D., and Dana R. CCR7 is critical for the induction and maintenance of Th17 immunity in dry eye disease. Invest. Ophthalmol. Vis. Sci. 55:5871–5877, 2014 25139737 10.1167/iovs.14-14481 PMC4168741

[49] Chen Y., Chauhan S.K., Lee H.S., Saban D.R., and Dana R. Chronic dry eye disease is principally mediated by effector memory Th17 cells. Mucosal Immunol. 7:38–45, 2014 23571503 10.1038/mi.2013.20 PMC3732510

[50] Pflugfelder S.C., Corrales R.M., and de Paiva C.S. T helper cytokines in dry eye disease. Exp. Eye Res. 117:118–125, 2013 24012834 10.1016/j.exer.2013.08.013 PMC3855838

[51] Mills K.H.G. Induction, function and regulation of IL-17-producing T cells. Eur. J. Immunol. 38:2636–2649, 2008 18958872 10.1002/eji.200838535

[52] Pflugfelder S.C., Stern M., Zhang S., and Shojaei A. LFA-1/ICAM-1 interaction as a therapeutic target in dry eye disease. J. Ocul. Pharmacol. Ther. 33:5–12, 2017 27906544 10.1089/jop.2016.0105 PMC5240001

[53] Rouzaut A., Garasa S., Teijeira, Á., González I., Martinez-Forero I., Suarez N., Larrea E., Alfaro C., Palazón A., Dubrot J., Hervás-Stubbs S., and Melero I. Dendritic cells adhere to and transmigrate across lymphatic endothelium in response to IFN-α. Eur. J. Immunol. 40:3054–3063, 2010 21061437 10.1002/eji.201040523

[54] Smith A., Stanley P., Jones K., Svensson L., McDowall A., and Hogg N. The role of the integrin LFA-1 in T-lymphocyte migration. Immunol. Rev. 218:135–146, 2007 17624950 10.1111/j.1600-065X.2007.00537.x

[55] Chauhan S.K., El Annan J., Ecoiffier T., Goyal S., Zhang Q., Saban D.R., and Dana R. Autoimmunity in dry eye is due to resistance of Th17 to Treg suppression. J. Immunol. 182:1247–1252, 2009 19155469 10.4049/jimmunol.182.3.1247 PMC2630586

[56] Chauhan S.K., Jin Y., Goyal S., Lee H.S., Fuchsluger T.A., Lee H.K., and Dana R. A novel pro-lymphangiogenic function for Th17/IL-17. Blood. 118:4630–4634, 2011 21908425 10.1182/blood-2011-01-332049 PMC3208279

[57] Ji Y.W., Lee J.L., Kang H.G., Gu N., Byun H., Yeo A., Noh H., Kim S., Choi E.Y., Song J.S., and Lee H.K. Corneal lymphangiogenesis facilitates ocular surface inflammation and cell trafficking in dry eye disease. Ocul. Surf. 16:306–313, 2018 29601983 10.1016/j.jtos.2018.03.008

[58] Dardalhon V., Korn T., Kuchroo V.K., and Anderson A.C. Role of Th1 and Th17 cells in organ-specific autoimmunity. J. Autoimmun. 31:252–256, 2008 18502610 10.1016/j.jaut.2008.04.017 PMC3178062

[59] Meloni M., Pauly A., Servi B.D., Varlet B.L., and Baudouin C. Occludin gene expression as an early *in vitro* sign for mild eye irritation assessment. Toxicol. In Vitro. 24:276–285, 2010 19729060 10.1016/j.tiv.2009.08.016

[60] De Paiva C.S., Chotikavanich S., Pangelinan S.B., Pitcher J.D. III, Fang B., Zheng X., Ma P., Farley W.J., Siemasko K.F., Niederkorn J.Y., Stern M.E., Li D.-Q., and Pflugfelder S.C. IL-17 disrupts corneal barrier following desiccating stress. Mucosal Immunol. 2:243–253, 2009 19242409 10.1038/mi.2009.5 PMC3594767

[61] Hogg N., Laschinger M., Giles K., and McDowall A. T-cell integrins: more than just sticking points. J. Cell Sci. 116:4695–4705, 2003 14600256 10.1242/jcs.00876

[62] Kürzinger K., and Springer T.A. Purification and structural characterization of LFA-1, a lymphocyte function-associated antigen, and Mac-1, a related macrophage differentiation antigen associated with the type three complement receptor. J. Biol. Chem. 257:12412–12418, 1982 6749860

[63] Lefort C.T., and Ley K. Neutrophil arrest by LFA-1 activation. Front. Immunol. 3:157, 2012 22701459 10.3389/fimmu.2012.00157 PMC3373145

[64] Gao J., Morgan G., Tieu D., Schwalb T.A., Luo J.Y., Wheeler L.A., and Stern M.E. ICAM-1 expression predisposes ocular tissues to immune-based inflammation in dry eye patients and Sjögrens syndrome-like MRL/lpr mice. Exp. Eye Res. 78:823–835, 2004 15037117 10.1016/j.exer.2003.10.024

[65] Min J.-K., Kim Y.-M., Kim S.W., Kwon M.-C., Kong Y.-Y., Hwang I.K., Won M.H., Rho J., and Kwon Y.-G. TNF-related activation-induced cytokine enhances leukocyte adhesiveness: induction of ICAM-1 and VCAM-1 via TNF receptor-associated factor and protein kinase C-dependent NF-κB activation in endothelial cells. J. Immunol. 175:531–540, 2005 15972689 10.4049/jimmunol.175.1.531

[66] Podgrabinska S., Kamalu O., Mayer L., Shimaoka M., Snoeck H., Randolph G.J., and Skobe M. Inflamed lymphatic endothelium suppresses dendritic cell maturation and function via Mac-1/ICAM-1-dependent mechanism. J. Immunol. 183:1767–1779, 2009 19587009 10.4049/jimmunol.0802167 PMC4410990

[67] Marlin S.D., and Springer T.A. Purified intercellular adhesion molecule-1 (ICAM-1) is a ligand for lymphocyte function-associated antigen 1 (LFA-1). Cell. 51:813–819, 1987 3315233 10.1016/0092-8674(87)90104-8

[68] Jones L., Downie L.E., Korb D., Benitez-del-Castillo J.M., Dana R., Deng S.X., Dong P.N., Geerling G., Hida R.Y., Liu Y., Seo K.Y., Tauber J., Wakamatsu T.H., Xu J., Wolffsohn J.S., and Craig J.P. TFOS DEWS II management and therapy report. Ocul. Surf. 15:575–628, 2017 28736343 10.1016/j.jtos.2017.05.006

[69] Guzmán M., Keitelman I., Sabbione F., Trevani A.S., Giordano M.N., and Galletti J.G. Desiccating stress-induced disruption of ocular surface immune tolerance drives dry eye disease. Clin. Exp. Immunol. 184:248–256, 2016 26690299 10.1111/cei.12759 PMC4837231

[70] Liden J., Rafter I., Truss M., Gustafsson J.-Å., and Okret S. Glucocorticoid effects on NF-κB binding in the transcription of the ICAM-1 gene. Biochem. Biophys. Res. Commun. 273:1008–1014, 2000 10891363 10.1006/bbrc.2000.3079

[71] Araki-Sasaki K., Katsuta O., Mano H., Nagano T., and Nakamura M. The effects of oral and topical corticosteroid in rabbit corneas. BMC Ophthalmol. 16:160, 2016 27596140 10.1186/s12886-016-0339-5 PMC5011848

[72] Marsh P., and Pflugfelder S.C. Topical nonpreserved methylprednisolone therapy for keratoconjunctivitis sicca in Sjögren syndrome. Ophthalmology. 106:811–816, 1999 10201607 10.1016/S0161-6420(99)90171-9

[73] Sheppard J.D., Toyos M.M., Kempen J.H., Kaur P., and Foster C.S. Difluprednate 0.05% versus prednisolone acetate 1% for endogenous anterior uveitis: a phase III, multicenter, randomized study. Invest. Ophthalmol. Vis. Sci. 55:2993–3002, 2014 24677110 10.1167/iovs.13-12660 PMC4581692

[74] Allergan. Restasis^®^ (cyclosporine ophthalmic emulsion) 0.05%. In: Allergan, ed. Allergan. Irvine, CA: Allergan; 2016

[75] Matsuda S., and Koyasu S. Mechanisms of action of cyclosporine. Immunopharmacology. 47:119–125, 2000 10878286 10.1016/s0162-3109(00)00192-2

[76] Kunert K.S., Tisdale A.S., Stern M.E., Smith J.A., and Gipson I.K. Analysis of topical cyclosporine treatment of patients with dry eye syndrome: effect on conjunctival lymphocytes. Arch. Ophthalmol. 118:1489–1496, 2000 11074805 10.1001/archopht.118.11.1489

[77] Strong B., Farley W., Stern M.E., and Pflugfelder S.C. Topical cyclosporine inhibits conjunctival epithelial apoptosis in experimental murine keratoconjunctivitis sicca. Cornea. 24:80–85, 2005 15604871 10.1097/01.ico.0000133994.22392.47

[78] Bose T.O., Pham Q.-M., Jellison E.R., Mouries J., Ballantyne C.M., and Lefrançois L. CD11a regulates effector CD8 T cell differentiation and central memory development in response to infection with *Listeria monocytogenes*. Infect. Immun. 81:1140–1151, 2013 23357382 10.1128/IAI.00749-12 PMC3639604

[79] Westera L., Drylewicz J., den Braber I., Mugwagwa T., van der Maas I., Kwast L., Volman T., van de Weg-Schrijver E.H., Bartha I., Spierenburg G., Gaiser K., Ackermans M.T., Asquith B., de Boer R.J., Tesselaar K., and Borghans J.A.M. Closing the gap between T-cell life span estimates from stable isotope-labeling studies in mice and humans. Blood. 122:2205–2212, 2013 23945154 10.1182/blood-2013-03-488411

[80] Sall K., Stevenson O.D., Mundorf T.K., and Reis B.L. Two multicenter, randomized studies of the efficacy and safety of cyclosporine ophthalmic emulsion in moderate to severe dry eye disease. Ophthalmology. 107:631–639, 2000 10768324 10.1016/s0161-6420(99)00176-1

[81] Mah F., Milner M., Yiu S., Donnenfeld E., Conway T.M., and Hollander D.A. PERSIST: physician's Evaluation of Restasis^®^ Satisfaction in Second Trial of topical cyclosporine ophthalmic emulsion 0.05% for dry eye: a retrospective review. Clin. Ophthalmol. 6:1971–1976, 2012 23226002 10.2147/OPTH.S30261 PMC3514052

[82] Luchs J. Phase 3 clinical results of cyclosporine 0.09% in a new nanomicellar ophthalmic solution to treatment keratoconjunctivitis sicca ASCRS-ASOA Annual Meeting. Washington, DC; 2018

[83] No authors listed. Lifitegrast (Xiidra) for dry eye disease. JAMA. 317:1473–1474, 2017 28399249 10.1001/jama.2016.12872

[84] Lollett I.V., and Galor A. Dry eye syndrome: developments and lifitegrast in perspective. Clin. Ophthalmol. 12:125–139, 2018 29391773 10.2147/OPTH.S126668 PMC5774475

[85] Murphy C.J., Bentley E., Miller P.E., McIntyre K., Leatherberry G., Dubielzig R., Giuliano E., Moore C.P., Phillips T.E., Smith P.B., Prescott E., Miller J.M., Thomas P., Scagliotti R., Esson D., Gadek T., and O'Neill C.A. The pharmacologic assessment of a novel lymphocyte function-associated antigen-1 antagonist (SAR 1118) for the treatment of keratoconjunctivitis sicca in dogs. Invest. Ophthalmol. Vis. Sci. 52:3174–3180, 2011 21330663 10.1167/iovs.09-5078

[86] Guimaraes de Souza R., Yu Z., Stern M.E., Pflugfelder S.C., and de Paiva C.S. Suppression of Th1-mediated keratoconjunctivitis sicca by lifitegrast. J. Ocul. Pharmacol. Ther. 34:543–549, 2018 29958030 10.1089/jop.2018.0047 PMC6909696

[87] Sun Y., Zhang R., Gadek T.R., O'Neill C.A., and Pearlman E. Corneal inflammation is inhibited by the LFA-1 antagonist, lifitegrast (SAR 1118). J. Ocul. Pharmacol. Ther. 29:395–402, 2013 23215542 10.1089/jop.2012.0102 PMC3643258

[88] Becker M.D., Garman K., Whitcup S.M., Planck S.R., and Rosenbaum J.T. Inhibition of leukocyte sticking and infiltration, but not rolling, by antibodies to ICAM-1 and LFA-1 in murine endotoxin–induced uveitis. Invest. Ophthalmol. Vis. Sci. 42:2563–2566, 2001 11581199

[89] Lam H., Bleiden L., de Paiva C.S., Farley W., Stern M.E., and Pflugfelder S.C. Tear cytokine profiles in dysfunctional tear syndrome. Am. J. Ophthalmol. 147:198–205, 2009 18992869 10.1016/j.ajo.2008.08.032 PMC3582020

[90] Reyes N.J., Yu C., Mathew R., Kunnen C.M., Kalnitsky J., Redfern R.L., Leonardi A., Perez V.L., MacLeod A.S., Gupta P.K., and Saban D.R. Neutrophils cause obstruction of eyelid sebaceous glands in inflammatory eye disease in mice. Sci. Transl. Med. 10, 2018 10.1126/scitranslmed.aas9164 PMC6597166 30045980

[91] Tauber J., Karpecki P., Latkany R., Luchs J., Martel J., Sall K., Raychaudhuri A., Smith V., and Semba C.P. Lifitegrast ophthalmic solution 5.0% versus placebo for treatment of dry eye disease: results of the randomized phase III OPUS-2 study. Ophthalmology. 122:2423–2431, 2015 26365210 10.1016/j.ophtha.2015.08.001

[92] Sheppard J.D., Torkildsen G.L., Lonsdale J.D., D'Ambrosio F.A. Jr., McLaurin E.B., Eiferman R.A., Kennedy K.S., and Semba C.P. Lifitegrast ophthalmic solution 5.0% for treatment of dry eye disease: results of the OPUS-1 phase 3 study. Ophthalmology. 121:475–483, 2014 24289915 10.1016/j.ophtha.2013.09.015

[93] Holland E.J., Luchs J., Karpecki P.M., Nichols K.K., Jackson M.A., Sall K., Tauber J., Roy M., Raychaudhuri A., and Shojaei A. Lifitegrast for the treatment of dry eye disease: results of a phase III, randomized, double-masked, placebo-controlled trial (OPUS-3). Ophthalmology. 124:53–60, 2017 28079022 10.1016/j.ophtha.2016.09.025

[94] Donnenfeld E.D., Karpecki P.M., Majmudar P.A., Nichols K.K., Raychaudhuri A., Roy M., and Semba C.P. Safety of lifitegrast ophthalmic solution 5.0% in patients with dry eye disease: a 1-year, multicenter, randomized, placebo-controlled study. Cornea. 35:741–748, 2016 27055211 10.1097/ICO.0000000000000803 PMC4859202

[95] Nichols K.K., Donnenfeld E.D., Karpecki P.M., Hovanesian J.A., Raychaudhuri A., Shojaei A., and Zhang S. Safety and tolerability of lifitegrast ophthalmic solution 5.0%: pooled analysis of five randomized controlled trials in dry eye disease. Eur. J. Ophthalmol. 1120672118791936, 2018 10.1177/1120672118791936 PMC6625033 30112930

[96] Kaur H., and Mutus B. Platelet function and thymosin β4. Biol. Chem. 393:595–598, 2012 22944663 10.1515/hsz-2012-0131

[97] Sosne G., and Ousler G.W. Thymosin beta 4 ophthalmic solution for dry eye: a randomized, placebo-controlled, phase II clinical trial conducted using the controlled adverse environment (CAE™) model. Clin. Ophthalmol. 9:877–884, 2015 26056426 10.2147/OPTH.S80954 PMC4445951

[98] Sosne G., Dunn S.P., and Kim C. Thymosin β4 significantly improves signs and symptoms of severe dry eye in a phase 2 randomized trial. Cornea. 34:491–496, 2015 25826322 10.1097/ICO.0000000000000379

[99] Sosne G., Kim C., and Kleinman H.K. Thymosin β4 significantly reduces the signs of dryness in a murine controlled adverse environment model of experimental dry eye. Expert Opin. Biol. Ther. 15 Suppl 1:S155–S161, 2015 26096547 10.1517/14712598.2015.1019858

[100] Sosne G., and Kleinman H.K. Primary mechanisms of thymosin β4 repair activity in dry eye disorders and other tissue injuries. Invest. Ophthalmol. Vis. Sci. 56:5110–5117, 2015 26241398 10.1167/iovs.15-16890

